# Future Innovations in Viral Immune Surveillance: A Novel Place for Bioinformation and Artificial Intelligence in the Administration of Health Care

**DOI:** 10.6026/97320630014201

**Published:** 2018-05-31

**Authors:** Francesco Chiappelli, Nicole Balenton, Allen Khakshooy

**Affiliations:** 1 UCLA Center for the Health Sciences, School of Dentistry, Los Angeles, CA; 2 CSUN Department of the Health Sciences, Northridge, CA; 3 UCLA School of Nursing; 4 Rappaport Faculty of Medicine, Technion-Israel Institute of Technology, Haifa, Israel 3109601

**Keywords:** Bioinformation, artificial intelligence, tweening, multiple regression, viral immune surveillance, Bunyavirus

## Abstract

Novel developments in bioinformation, bioinformatics and biostatistics, including artificial intelligence (AI), play a timely and critical role in translational care. Case in point, the extent to which viral immune surveillance is regulated by immune cells and soluble factors, and by non-immune factors informs the administration of health care. The events by which health is regained following viral infection is an allostatic process, which can be modeled using Hilbert's and Volterra's mathematical biology criteria, and biostatistical methodologies such as linear multiple regression. Health regained following viral infection can be given as Y being the sum-total of the positive factors and events (∏) that inherently push allostasis forward (i.e., the orderly process of immune activation and maturation) and the negative (N) factors and events that, allostatically speaking, interfere with regaining health. Any gaps in knowledge are filled by AI-aided immune tweening. Proof of concept can be tested with the fast-gaining infection using tick-borne Bunyavirus that cause severe fever with thrombocytopenia syndrome (SFTS).

## Background

Recent progress in the administration of health care has identified two principal facets: translational research, the generation of fundamental research evidence about the underlying pathobiology for implementation in patient care, and translational effectiveness, the consensus of the best available evidence for clinical intervention. Bioinformation, bioinformatics and biostatistics also play a timely and critical role in translational care [1, 2].

Case in point, as the primary host defense system against viral infections, the immune system is comprised of several soluble and insoluble components segregated as innate and acquired immunity. T lymphocytes that express the cluster differentiation # 3 (CD3) and the alpha/beta form of the T cell receptor (TcR) play a critical role in anti-viral cellular immune surveillance. T cells recognize non-self in association with the major histocompatibility complex (MHC). Class I MHC on host cells presents non-self-peptides that derive from internal antigens, including tumor markers of viral genomes integrated into the host DNA. MHC Class II on antigen-presenting cells (i.e., macrophages, dendritic cells) presents non-self-peptides derived from the breakdown of bacterial and parasitic foreign pathogens. MHC Class I only trigger the activation of CD3/TcR cells that express the CD8 moiety, and MHC Class II trigger CD3/TcR cells that express CD4 [3, 4, 5, 6].

The presentation of antigenic non-self via MHC to either CD8+ or CD4+ T cells initiates the process of activation, proliferation and terminal maturation. Together, these events ensure a complete, effective and efficient immune surveillance against viral infection, and can be monitored by functional and phenotypic studies. These stages have been well recognized, extensively studied and characterized in detail in the last decades. They involve soluble immune components, such as, initially, the production of the T cell growth factor, known as interleukin (IL)- 2, and the expression on the T cell membrane of the alpha chain, CD25, of the IL-2 receptor - thus, a CD25+ T cell is one that is fully activated and actively engaged in multiplication. As the activated T cell divides, several proliferation markers can be detected, including cysteine-aspartic proteases, cysteine aspartases or cysteine-dependent aspartate-directed proteases (i.e., Caspases). During this replicative period, the restriction fragment A of the common leukocyte antigen, CD45, is lost, and the much shorter 0 fragment is expressed on the lymphocyte membrane. This transition from CD3/TcR+CD8+CD45RA+ to CD3/TcR+CD8+CD45R0+ indicates the terminal maturation process from an antigen-naive to a memory T cell [3, 4, 5, 6].

Both the CD4+ and CD8+ sub-population is endowed on naive (CD45RA+) and memory (CD45R0+), resting (CD25-) and activated (CD25+) cells that express the genomic marker FoxP3. These cells are regulatory T cells (TRegs) in that they attenuate T cell-mediated immune responses. By far the larger proportion of TRegs is recognized by the characteristic immunophenotypic profile of CD45RA+CD3+CD4+CD25+Foxp3+ [3, 4, 5, 6].

Taken together, CD4 T cells that are primarily engaged in producing the cytokines required for concerted immune surveillance (e.g., TH1 and TH2 cytokine profiles), CD8 T cells that are primarily cytotoxic killing virally-infected cells, and Tregs coordinate the finely tuned cell-mediated immune surveillance events. Cytokines (e.g., IL's, interferons) are among the large family of soluble immune factors that contribute to the regulation of cell-mediated immune events [3, 4, 5, 6].

Non-immune factors (e.g., neuropeptides, hormones) and cells (e.g., sympathetic and parasympathetic innervation, cranial nerves), and other psychophysiological processes (e.g., nutrition, sleep, anxiety, stress) provide significant modulation of cellmediated viral immune surveillance. Related physiological processes (e.g., bone metabolism, osteology) are also intertwined with immunological responses. Together, psychoneuroendocrine and osteological cross-regulation of cellular immunity opened the novel field of psychoneuroendocrineosteoimmunology or as we have previously introduced as the psychoneuroendocrine osteoimmune interactome [7, 8, 9, 10, 11, 17]

Together, traditional canonical immune and psychoneuroendocrineosteoimmune regulatory mechanisms converge to maintain the organism in a state of physiological balance - i.e., health - which physiologists call homeostasis. Exposure to a pathogen produces a state of un-health, or disease ensues, which physiologically is characterized as imbalance, and termed heterostasis. The spectrum of events outlined above that involve both canonical immune responses and the complex array of psychoneuroendocrineosteoimmune regulation of these events drive the organisms to the physiological equilibrium of homeostasis, which is health regained. The return from heterostasis to homeostasis is a process that physiologists term allostasis [8, 9, 10]

With respect to viral immune surveillance, viral infection challenges immune homeostasis and leads virally induced heterostasis. The concerted canonical immune and psychoneuroendocrineosteoimmune events come together to control the pathophysiology that follows viral infection correspond to the process of immune allostasis. When the pathobiology of the viral infection is resolved (e.g., rhinovirus [common cold]), then the process is said to have been immune allostasis type I. On the contrary, when the pathobiology of the viral infection is chronic (e.g., retroviruses, such as HIV and Zika, whose genomes remain incorporated in the host's DNA), then process is an immune allostasis type II.

From the perspective of biomedical modelling, it will behoove the administration of health care for virally infected patients to consider the desired outcome, Y, as the state of regained homeostasis; in other words, health. Y, the homeostatic state of health regained following a viral infection, is simply the sum of positive and negative factors and/or events. Positive factors and events (∏) inherently push allostasis forward (i.e., the orderly process of immune activation and maturation). On the other hand, negative (N) factors and events, allostatically speaking, interfere with attaining Y. Collectively, Y represents the sumproduct of the fine, coordinated and time-regulated interaction of the interacting ∏'s and N's during anti-viral immune surveillance. Thus, Y, the state of regained health, is given as (Y=∑∏ + ∑N).

## Methodology

The question then becomes, knowing what we know today about the constituents of ∑∏ and of ∑N, can we not design, by means of bioinformatics, artificial ∏'s and N's, that may push the organism's response more securely through all the allostatic phases to Y, the homeostatic state of health regained following a viral infection? Physiology has been able to a related feat by producing bioinformatics particles, which when injected in patients help improve both diagnostics and treatment (e.g., regulation of cholesterol levels).

Future artificial intelligence (AI) advances will produce the artificial ∏'s and N's, which will aid regaining Y. In the meanwhile, as science continues to complete our knowledge of all the ∏'s and N's involved, "tweening" is the computerized process that aids uncovering the end product of a sequence. In this case, the steps in-between to be programmed, and be applied to our conceptualization of immune surveillance events fall into a novel conceptualization of AI-aided tweening, which we propose to be called "immune tweening".

The statistical process of regression was first developed by Sir Francis Galton (1822-1911) to describe certain biological phenomena, such as predicting that the heights of descendants of tall ancestors tend to regress down towards a normal average. The inherent behavior of all biological processes to regress toward the mean is often described as the concept of central tendency. In time, Galton's original conceptualization of regression as describing biological phenomena was expanded into related sciences, including psychobiology and educational psychology. Pearson, one of his students, and Yule, one of Pearson's students extended the use of regression more widely to a more general statistical context. It is befitting that we propose here to return to Galton's original purpose of regression to embrace the biological phenomenon of viral immunity.

In brief, statistical modeling describes simple or multiple linear or nonlinear regressions as a set of statistical processes for estimating the relationships among variables. Specifically, regression serves to predict an outcome Y, based on quantifiable predictor variables. Regression analysis contributes to the understanding of how the typical value of the dependent variable changes when any one of the independent variables is varied, while the other independent variables are held fixed. Stated somewhat differently, regression is a biostatistical tool that estimates the conditional expectation of the dependent variable, Y, given quantifiable values of independent variables. The function of the independent variables is the regression function to be estimated, which when resolved quantifies Y, the outcome variable [1, 2, 12].

Taken together, the application of this biostatistical technique to an organism's allostatic processes makes the problem a relatively easy-to-use multiple regression, Y=∑∏ + ∑N + ε. Where, ε being the residual random error.

In this simple multiple regression model, which for all intents and purposes can be assumed to be linear, the outcome (Y) is the summation of positive variables that push allostasis forward (∑∏) and the sum-total of negative variables (∑N) that interfere or dampen allostasis. The third variable of the equation, namely, the residual random error (ε), represents the unexplainable deviations that result from individual differences relative to an organism state of homeostasis following viral infection. It is simple to consider the existence of factors and or events that fall neither in the positive nor negative variables. Thus, as this computerized system develops, the elucidation of more specific factors impacting regained homeostasis will lead to the implementation of a seminal biostatistical technique referred to as error fractionation. This, in turn, will lead to a more complex prediction model and a reduction of unexplainable or residual random error.

The model allows us to predict quantifiably the extent to which the outcome variable of health regained (that is of recovered homeostasis) following a viral infection is effected when either of the variables is changed, while holding the other constant. The prediction will be interpreted as the fine, coordinated, and timeregulated processes that occur throughout all the relevant interacting variables during immune surveillance. The residual random error can be further fractionated by hierarchically expanding the model, as our understanding of immunophysiology fills the remaining gaps in knowledge, which will be temporarily filled by the process of AI-aided immune tweening.

## Discussion

Professor David Hilbert (1862-1943) made a profound impact on our perspective and ability to face certain key global health problems by the utilization of mathematics. An ardent defender of the belief that all the mysteries of biology will eventually be uncovered for the betterment of human health, he stated in his retirement address to the Society of German Scientists and Physicians, on 8 September 1930: Wir mussen wissen. Wir werden wissen (we must know. We will know). That statement was a direct response to his contemporary, Emil Heinrich du Bois- Reymond (1818-1896) who had written, in his 1872 Uber die Grenzen des Naturerkennens (On the limits of our understanding of nature), Ignoramus et ignorabimus ([there are things] we do not know and we shall not know).

Hilbert's timely and critical contributions inspired Vito Volterra (1860-1940) and others in proposing, testing and establishing functional analysis and its implications and applications to mathematical biology (cf., Lokta-Volterra equations for the modeling of the epidemiology of infectious diseases). Current evidence-based advances might most likely lead Hilbert to respond to du Bois-Reymond in even more specific and assertive terms, such as Quod ignoramus sciturum est (what we do not know [now] will [certainly] be made uncovered). Today's advances in translational science [1, 2] are indebted to Hilbert.

Innovations in immunization data management, use, and improved process efficiency are timely and critical as potentially fatal viral diseases; old [13, 14] and new threats the human population globally. Immunization is of the most valuable and cost-effective public health interventions. It is estimated that millions of lives are spared using concerted vaccination programs globally on an annual basis. Vaccination contributes to healthy child development and improved quality of life among adults worldwide: in fact, recent epidemiological estimates suggest a significant return on investment (ROI) of immunization programs - every 1 USD invested in immunization leads to 15-50 USD in health economic benefits.

Important gaps in knowledge remain in our ability to fully harness the potential value of immunization data in informing program management. Expectations are that AI and AI-aided tweening will promote the effective use of timely and relevant data to drive programmatic performance of immunization to new and old viral threats. The incorporation basic translational research knowledge base, of Hilbert's and Volterra's mathematical, of biostatistical linear multiple regression, be it using probabilistic Fisherian or Bayesian inference modeling, and of immune tweening, will generate new, efficacious, effective and efficient concerted vaccination programs globally. Expectations are that AI and bioinformation will contribute successfully to enhancing the effectiveness of health care administration by identifying and securing the timely utilization of the most relevant data to dramatically improve the performance, and secure the success of viral immunization programs globally.

To demonstrate the proof of concept in principle and the feasibility of the novel conceptualization of viral immune surveillance proposed here, and to establish its practical potential novel research avenues in bioinformation ought to address the problem of a new, emerging and potentially grave viral outbreak, which, if left unchecked, could, in the matter of a few years, be as critical and severe as recent epidemiological crises of viral outbreaks [3, 7, 13, 14]. The virus causing the severe fever with thrombocytopenia syndrome (SFTS), a viral disease first reported in China in 2009, and fast spreading in South-East Asia, could be a useful model for that purpose.

SFTS, a fulminant potentially fatal tick-borne virus (Phlebovirus, Bunyavirus family, negative-sense single-stranded enveloped RNA virus, transmitted by arthropods), is related to Hantaviruses, and may prove to have similar characteristics of incidence of infection, morbidity and mortality. SFTS leads to a myriad of symptoms, including fever, fatigue, chill, headache, lymphadenopathy, thrombocytopenia, leukocytopenia, anorexia, nausea, myalgia, diarrhea, vomiting, abdominal pain, gingival hemorrhage, conjunctival congestion, and, like Ebola, lifethreatening hemorrhagic fevers. SFTS is highly pathogenic, and severe cases are fatal. Epidemics can be prolonged, and fears of pandemics are fueled by the reports that infections can be transmitted to humans as well by the bites of infected pets.

SFTS is transmitted using ticks that infect livestock, and consequentially SFTS outbreaks tend to involve adults and children who live in farmlands, in regular contact with animals, and involved work in agriculture or forestry. The SFTS-carrying tick - predominantly the ixodid tick species - tends to breed at warmer temperature and in more southerly regions. Indeed, arthropodological evidence indicates that the ixodid ticks tend to adapt to climate change, and invade northwards as the temperature rises. Epidemiological data indicate that SFTS infection rates are fast increasing SFTS has not been reported in US territories (e.g., Guam, Puerto Rico) or the mainland, but the lessons of HIV, Ebola and Zika are vivid.

Protocol-wise, the proposed model requires establishing a working relationship with a WHO Regional Office, collecting and systematically entering epidemiological and individual patient data for analysis, and testing a mathematical Hilbertian- Volterrian biostatistical multiple regression model. The outcome variable Y, immunity regained following SFTS infection, will be derived from immune and psychobiological predictors of the model outlined above. The raw data used to refine the regression model will be the data provided by the WHO Regional Office. Immune tweening will be a critical AI computerized and bioinformation-driven process to identify and program all the necessary steps required for effective immunization and immunization data delivery. In a second phase, Hilbertian- Volterrian biostatistical multiple regression model of immunization will be generalized to Dengue, Ebola and Zika globally.

## Conclusion

The future of immunity lies in our cogent utilization of AI, and specifically bioinformatics to generate psycho-neuroendocrineosteo- immune algorithms, completed by immune-tweening, to predict the successful outcome of the allostatic process of immune response to a viral pathogen. This can be envisaged to apply to a variety of viral antigens, such as HIV, or related retroviruses including Zika and Ebola, or new and aggressive variants of orthomyxoviruses, such as the various genera of influenza virus (isavirus, thogotovirus and quaranjavirus, and influenza virus genus A, B, C, and D, with genus A being the most dangerous to humans for causing influenza). There are several subtypes of influenza A viruses, which all can infect humans with a broad spectrum of severity. They are classified, based on the viral surface proteins hemagglutinin (HA or H) and neuraminidase (NA or N). Sixteen H subtypes and nine N subtypes of influenza a virus have been identified to this date, but active virology research uncovers more H and N subtypes continuously. Type A influenza subtypes, determined by their H & N signatures are the most virulent human pathogens and cause most severe, often fulminant and lethal disease. Suffice to remember, the Spanish flu and the Swine flu (H1N1), the Asian flu (H2N2), the pandemic flu threat (H5N1) and the like in the last century alone.

Well-informed bioinformatics, advanced systems immunology databases (e.g., Genbank, UniProt, InterPro, Pfam, KEGG, BioCyc, GenoCAD, Bcipep), comparative orthology analysis (i.e., the study of the physiological and functional correspondence among pertinent genes, and interactomic structures), statistical techniques such as multiple regression as outlined above, and carefully derived psycho-neuroendocrine-osteo-immune algorithms (e.g., k-means and hierarchical clustering; network analysis; high-throughput and high-fidelity quantification of fractal dimension analysis and sub-cellular localization, and related bioimage-informatics), and appropriate immunetweening will, in the near future, lead us to describe and characterize the success of the allostatic process of immune response to these viral pathogens, and any immunogens, to a state of immunophysiological recovery, Y.

In short, AI and virtual reality promise to be most useful in the administration of health care in general, and particularly with respect to viral immunity in many ways. Moreover, AI will open new avenues of translational research, and improved cost and benefit effectiveness [15]. The prediction model we have proposed [16] defends that AI will promote the establishment of the novel science of immune-tweening, which will help us understand and complete a set of immune and non-immune events that lead to immunity to a viral threat. AI-aided and bioinformation-driven will provide novel giant steps toward the effective use of timely and relevant data to drive programmatic performance specifically of immunization to new and old viral threats, and for the administration of health care in general. These new developments will aid face new threats of viral epidemics, including SFTS and other members of the Bunyavirus family.
